# Challenges of Long-Gap Esophageal Atresia Repair in a Patient With Tetralogy of Fallot and Right Aortic Arch

**DOI:** 10.7759/cureus.90412

**Published:** 2025-08-18

**Authors:** Ewa A Bieganska, Marek Wolski

**Affiliations:** 1 Department of Paediatric Surgery, Medical University of Warsaw, Warsaw, POL

**Keywords:** long-gap esophageal atresia, pediatric thoracoscopy, right aortic arch (raa), tetrology of fallot, thoracoscopic internal traction

## Abstract

In cases of complex vascular anomalies or rare variants of esophageal atresia (EA), such as long-gap variant, individualized surgical planning becomes even more critical. We present a case report of a patient with long-gap EA, tetralogy of fallot (ToF), and right aortic arch (RAA), in which an initial right thoracotomy was unsuccessful due to unexpected vascular anatomy. Subsequent computed tomography (CT) imaging revealed the precise vascular configuration, prompting a change to a left thoracoscopic approach and staged repair using internal traction followed by early anastomosis. This case underscores the importance of preoperative vascular assessment and highlights the need for flexible surgical strategies in complex EA cases.

## Introduction

Esophageal atresia (EA) is the most common congenital defect of the esophagus, and occurs in approximately two to four cases per 10,000 live births [1,2]. Substantial percentage (around 30%-50%) of patients with EA have accompanying malformations, with about 10% fulfilling criteria for syndrome or association, like VACTERL (vertebral anomalies, anorectal anomalies, cardiac anomalies, tracheoesophageal fistula or atresia, renal anomalies, and limb anomalies) or CHARGE (coloboma, heart defects, atresia of the choanae (nasal passages), retardation of growth and development, genital abnormalities, and ear abnormalities) [2-4]. Among these malformations, cardiovascular anomalies are one of the most frequently associated with EA and also one of the most relevant for the complication of the surgical field during defect correction [5]. The presence of right aortic arch (RAA), which is estimated in about 2%-5% of the cases, significantly alters the mediastinal anatomy, often displacing the trachea and esophageal pouches toward the left hemithorax. This, in turn, motivates surgeons to consider alternative surgical access routes to the classic approach - right thoracotomy [5-7].

Despite pre-surgical echocardiography now being routinely performed, RAA is frequently undiagnosed before surgery. Some studies report that as few as 20% of cases of RAA are correctly identified [5,8,9]. Given this and the rarity of such patients, the optimal surgical strategy for these cases remains a matter of debate.

The situation is further complicated by rarer forms of EA, such as long-gap atresia. As this form of the condition accounts for only around 10% of the cases, the optimal surgical management is also subject to some controversy [10]. Methods range from delayed thoracoscopic anastomosis, advocated by S. S. Rothenberg, to staged internal traction with anastomosis in early weeks of patient’s life, developed by D. Patkowski [11-13]. Despite these differing approaches, however, all agree that this condition poses a significant surgical challenge.

## Case presentation

We present a case of a male neonate born in good general condition at 35 weeks of gestation, from fourth pregnancy and third delivery, with a birth weight of 2050 g (15th percentile). Prenatally, he was diagnosed with tetralogy of fallot (ToF), which included severe stenosis of the right ventricular outflow tract and major aortopulmonary collateral arteries (MAPCAs). Additionally, a pelvic kidney was suspected. No other abnormalities were found during prenatal diagnostics. The course of the pregnancy was complicated by gestational diabetes mellitus, which was managed by diet. The mother had also von Willebrand disease and was hepatitis B surface antibody (HBs) positive.

Physical examination performed just after birth revealed a systolic murmur of 2/6 on the Levine scale, anal atresia, skin features of prematurity and central cyanosis, a possible submucosal cleft of the secondary palate, and discrete dysmorphic features of the face (a low-set hairline, low-set ears, and a fairly wide nasal base).

**Figure 1:**
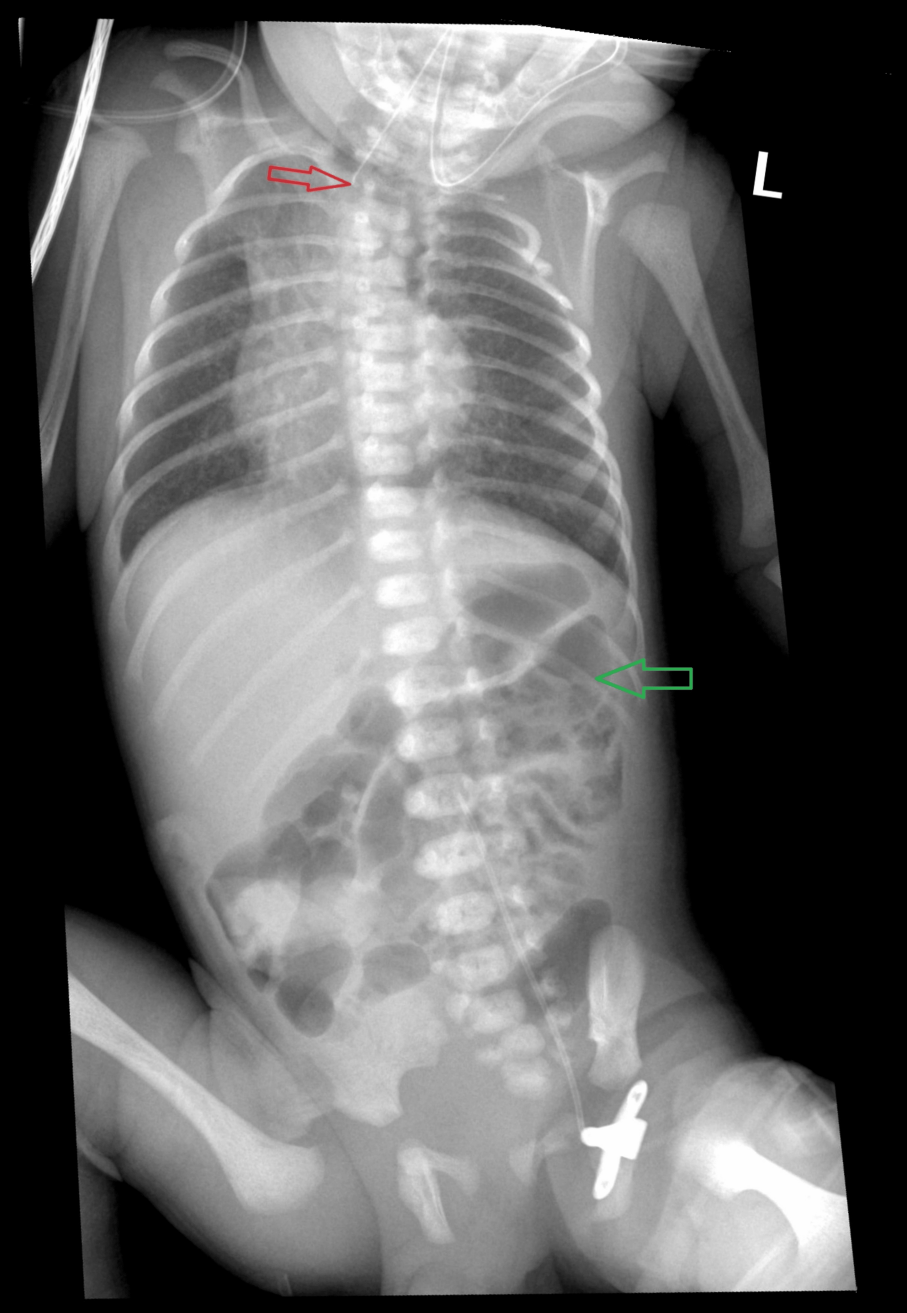
The after-birth chest and abdomen X-ray The red arrow points to the end of the gastric probe at cervical 7 /thoracic 1( C7/Th1 ) level. The green arrow points to the air in intestinal loops.

When attempting to insert a gastric probe, resistance was encountered at 7 cm. Chest X-ray was performed (Figure 1), which revealed the end of the probe at C7/Th1 level. It is also worth noting that the X-ray showed aerated intestinal loops, which, according to Gross's classification, may suggest the presence of type C oesophageal atresia. In addition, vertebral defects in the form of butterfly vertebral morphology of the Th2 and Th4 vertebrae, cervical ribs, and rib defects were revealed.

On abdominal ultrasound, the left kidney was localized slightly lower than normal, and the rectum was revealed ending about 10 mm from the skin surface, with blind anal canal.

The post-natal echocardiography confirmed ToF, pulmonary valve stenosis, right-sided aortic arch, wandering left subclavian artery, MAPCAs, and persistent left superior vena cava with coronary sinus (L-SVC-Cs). Additionally, the absence of the thymus was shown.

Based on the above results, the patient was diagnosed with VACTERL association. Due to repeated decreases in blood oxygenation in the postnatal period, the patient was intubated and mechanical ventilation was initiated. After the confirmation of cardiac abnormality, alprostadil infusion at a dose of 0.01 μg/kg/min was administered and continued until day 7 of life.

The patient's treatment plan was established by a multispecialist team due to the presence of multiple congenital malformations. The decision was made to begin treatment by addressing the gastrointestinal defects, followed by cardiac management in the form of diagnostic cardiac catheterization, and an attempt to unblock and expand the pulmonary artery.

The first operation was performed on day 2 of the patient’s life. Under general anaesthesia, a direct bronchoscopy was performed to inspect the trachea. No tracheomalacia was observed, but a lower fistula was seen on the posterior wall of the trachea at the point of bifurcation. Otherwise, the division into the main bronchi was normal. No upper fistula was visualized.

The patient was then positioned on the left side. A posterior-lateral right-sided thoracotomy was performed with access through the third intercostal space. The inferior fistula was dissected and ligated, then cut off right next to the trachea. In the region of the posterior superior mediastinum, the aortic arch was visualized, with the subclavian artery obscuring the upper thoracic entry. The aorta and subclavian artery were dissected and the aorta was elevated using tape. The upper part of the oesophagus could not be visualized, even after inserting the gastric tube, which effectively led to the diagnosis of long-gap esophageal atresia (LGEA). Due to the patient's unstable condition, further preparation of the upper esophageal stump was abandoned. The chest wall was closed. Subsequently, a loop stoma on the descending colon was created during the same procedure.

**Figure 2:**
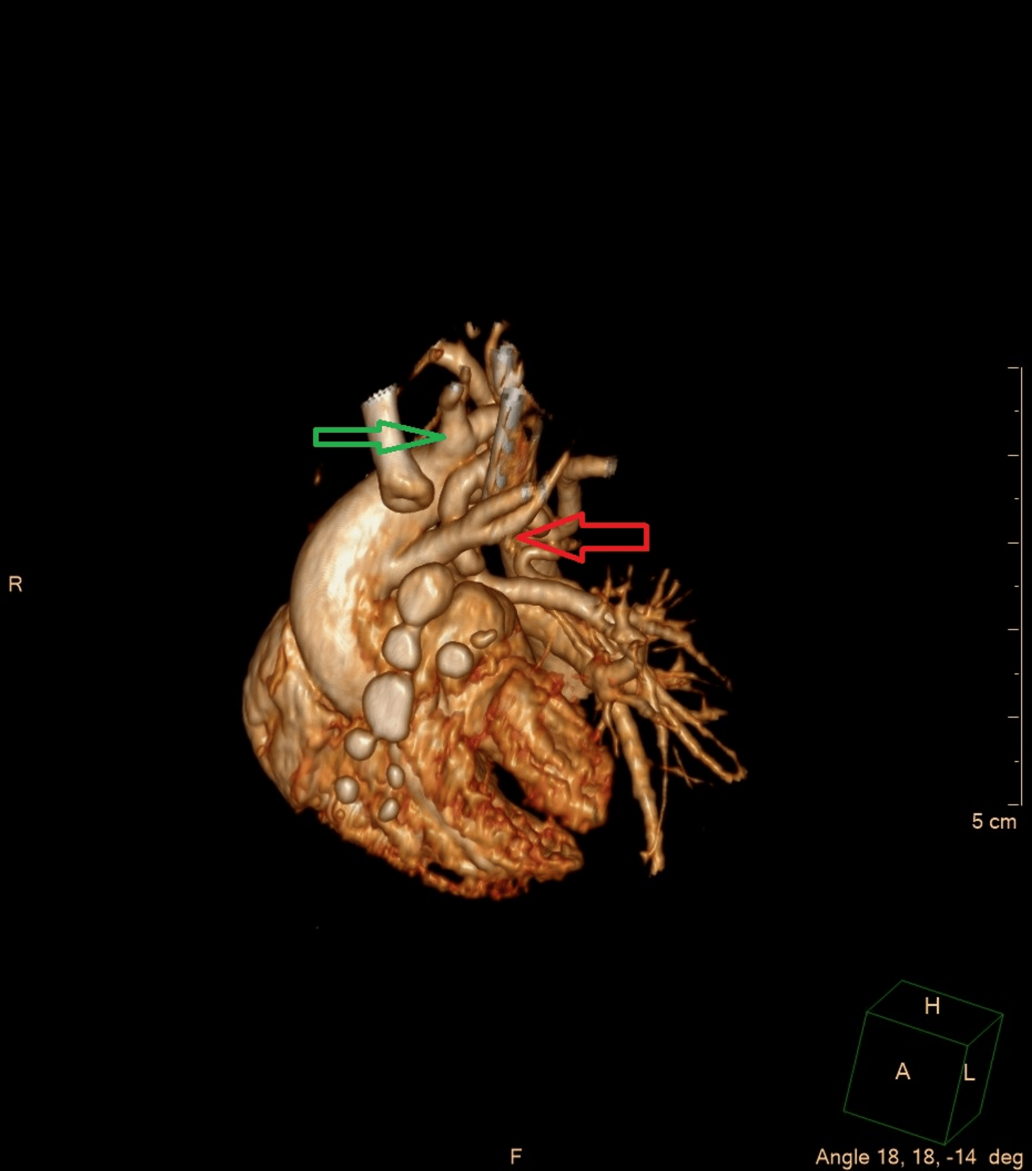
Image from the heart reconstruction created basing on the CT scans (volume-rendered images); front projection The red arrow points to the left common carotid artery, with an early division of the external and internal carotid arteries. The green arrow points to the right common carotid artery. Image credit: Created by the Pediatric Radiology Department team at the University Clinical Center of the Medical University of Warsaw.

**Figure 3:**
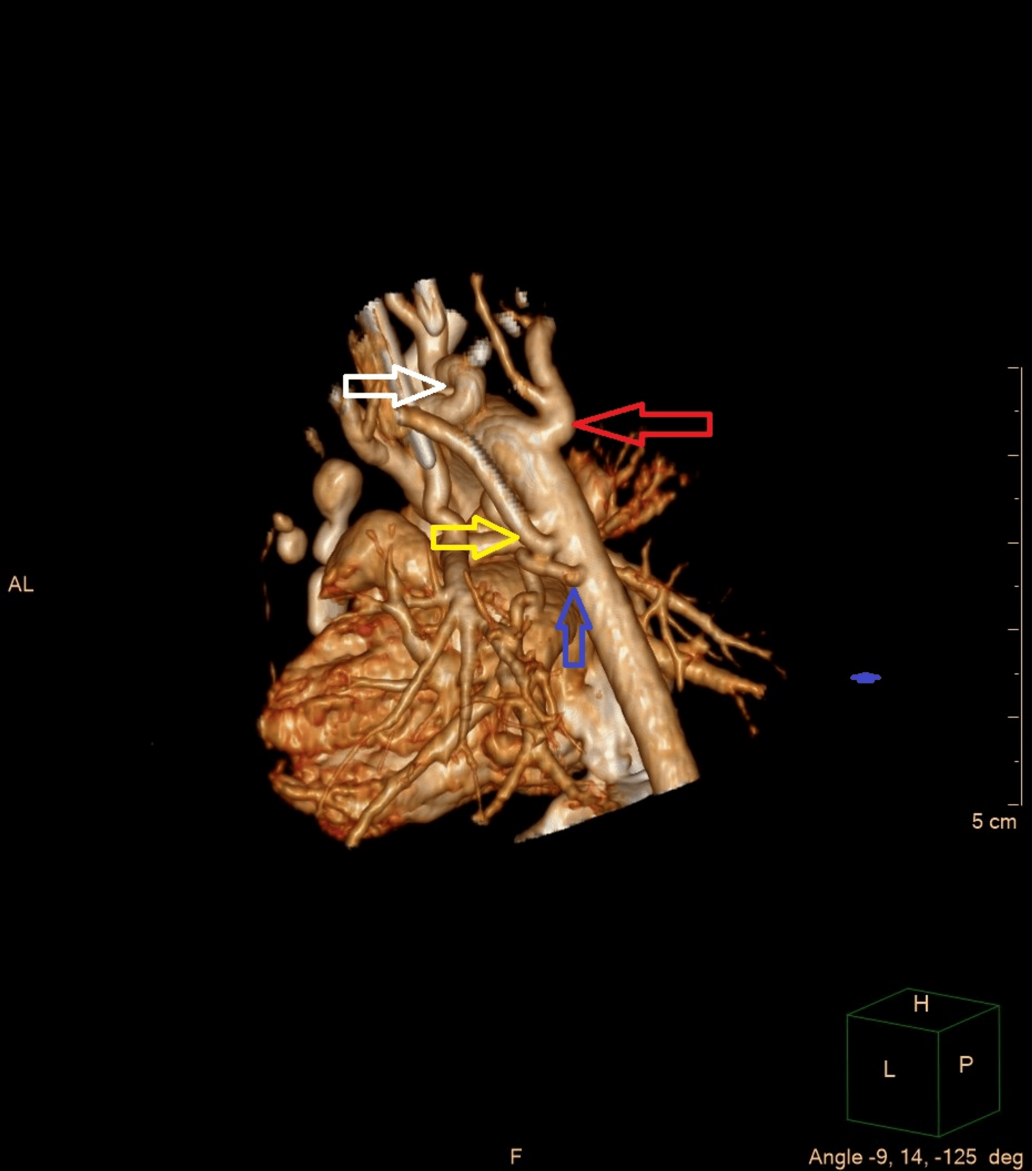
Images from the heart reconstruction created basing on the CT scans (volume-rendered images); back projection The white arrow points to the ductus arteriosus; the red arrow points to the right vertebral artery; the yellow arrow points to the left subclavian artery; the blue arrow points to the one of the MAPCAs. Image credit: Created by the Pediatric Radiology Department team at the University Clinical Center of the Medical University of Warsaw.

Following the initial surgical procedure, a cardiac CT scan was performed to assess the exact anatomy. The examination revealed the following: a right-sided aortic arch; the left subclavian artery descending from the descending aorta after the right vertebral artery (wandering left subclavian artery); wide MAPCAs up to 2.5 mm in diameter were present at the level of the subclavian artery and below. Images from the reconstruction created based on the CT scans are presented in Figures 2, 3.

On day 10 of the patient's life, pulmonary haemorrhage occurred, and it was decided to perform cardiac catheterization and close one of the collateral circulation vessels supplying the lungs, as well as perform balloon valvuloplasty of the pulmonary valve. Following the procedure, the child's clinical condition improved and symptoms of pulmonary haemorrhage disappeared.

The cardiac picture remained stable in regularly performed echocardiography. The patient received diuretic treatment with spironolactone and furosemide.

**Figure 4:**
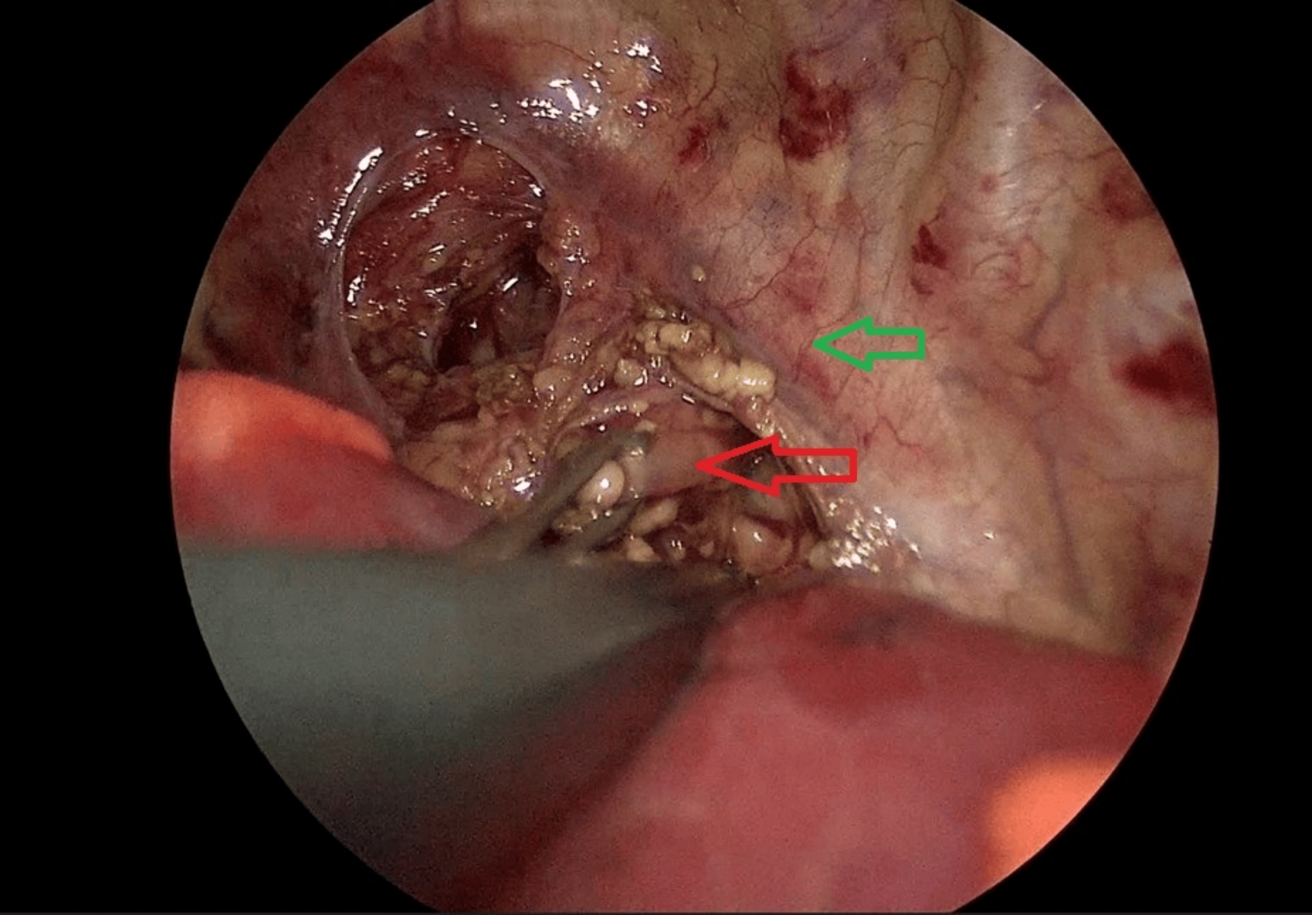
Image from thoracoscopy. The upper esophageal stump is retracted after dissection. The red arrow points to the upper esophageal stump. The green arrow points to the vertebral column.

**Figure 5:**
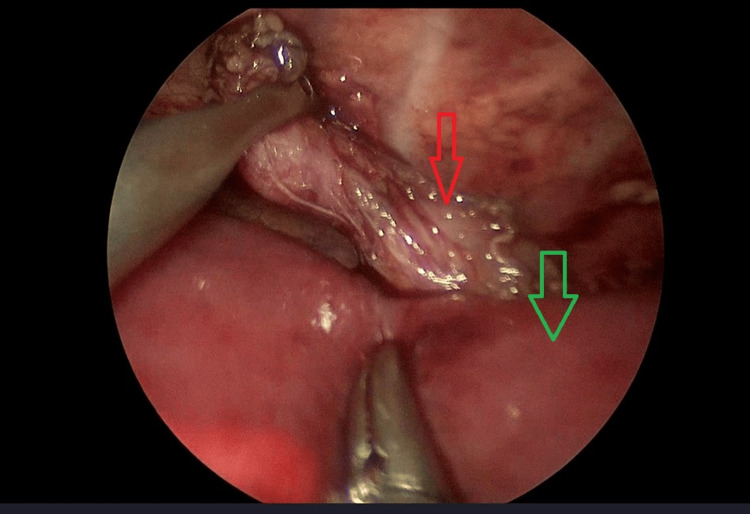
Image from thoracoscopy. Lower esophageal stump after dissection. The red arrow points to the lower esophageal stump. The green arrow points to the left lung.

Approximately two weeks after the initial surgery, a gastrostomy was performed to enable gastrointestinal feeding. Around five weeks after the initial operation, during which the lower fistula was ligated, a second operation was performed in an attempt to create an esophageal anastomosis or install internal traction. Due to the patient's abnormal vascular anatomy confirmed in diagnostic imaging, it was decided to take a left-sided thoracoscopic approach. To improve visualization of the lower esophageal stump, a Foley catheter with an indocyanine (ICG) green-filled balloon was inserted into the esophagus via a gastroscope before thoracoscopy. The patient was then repositioned, with trocars placed in the posterior axillary line at the angle of the scapula, as well as two 3.5 mm trocars anteriorly, one above and one below the initial trocar. The upper esophageal stump was then dissected from the trachea (Figures 4, 5).

**Figure 6:**
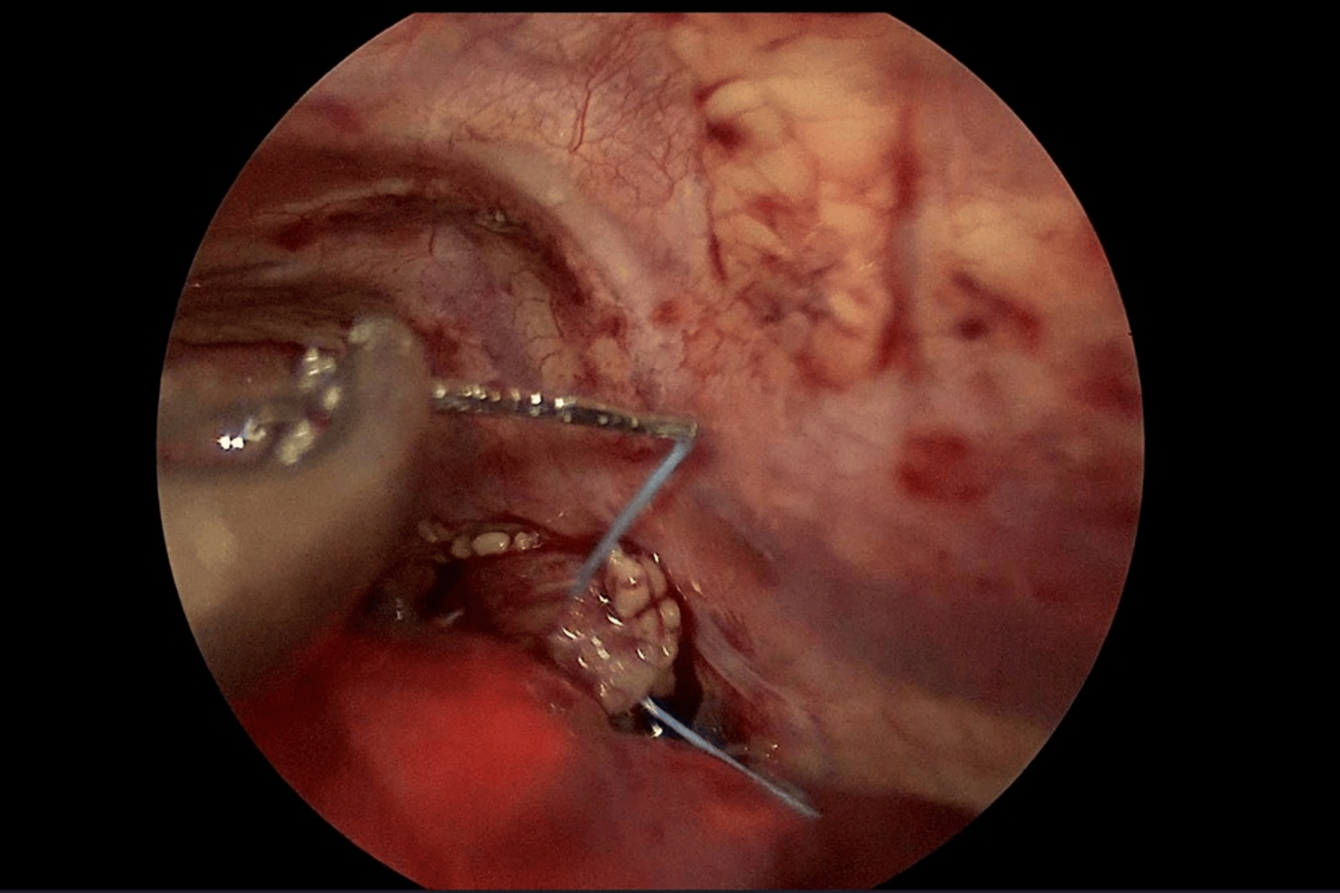
Image from thoracoscopy. A traction suture has been applied to the upper stump.

**Figure 7:**
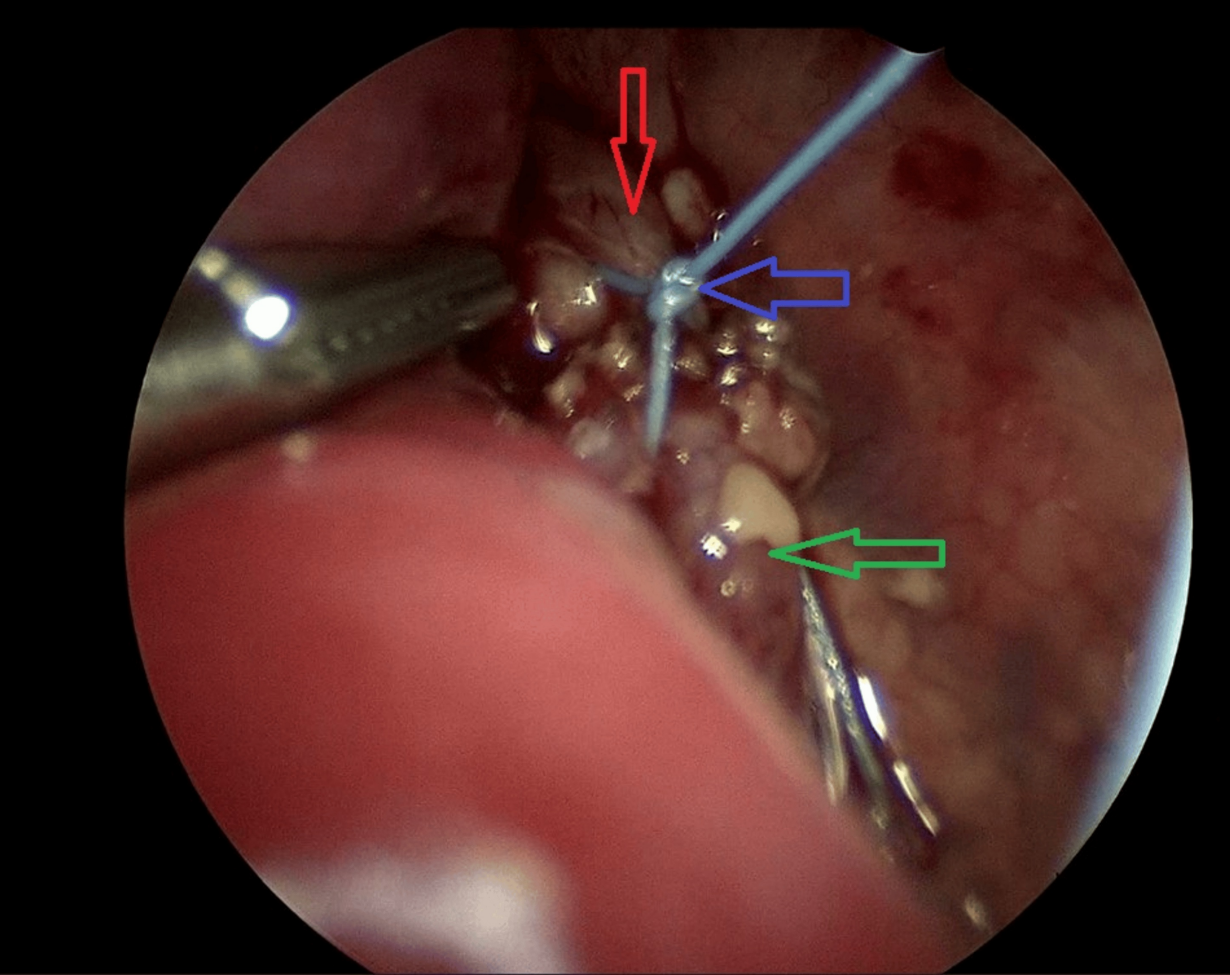
Image from thoracoscopy. The final position of stumps after approximation. The red arrow points to the upper esophageal stump. The green arrow points to the lower esophageal stump. The blue arrow points to the knot approximating the stumps.

After starting the preparation of the lower esophageal stump, the ICG-filled balloon unfortunately did not become visible. Nevertheless, the dissection from the surrounding tissues and adhesions was successful, and the two ends were joined together with a traction suture under medium tension (Figures 6, 7).

One week after the internal traction was inserted, another thoracoscopy was performed to create an esophageal anastomosis or reinsert the traction. As in the previous procedure, a 5 mm optic and a 3 mm working trocar were placed in the thorax on the left side. Both parts of the esophagus were visualized and dissected from the surrounding tissues. The length of the esophagus was assessed as sufficient for anastomosis. However, due to the challenging surgical conditions and slow progress of the procedure, it was decided to convert to a left posterolateral thoracotomy. The esophagus was anastomosed under high tension. A Flocare 6.0 probe was left in the stomach, as was a 12 Fr pleural drainage. As the anastomosis was performed under high tension, it was decided to leave the Replogle probe in the proximal part of the esophagus during the postoperative period. The postoperative period was uneventful. Two weeks after esophageal anastomosis, contrast studies were performed and no leakage was found.

Unfortunately, the patient died on day 93 of life as a result of increased airway obstruction that was unresponsive to treatment.

## Discussion

Although cardiovascular anomalies, including ToF and defects of great arteries, are the most common birth defects accompanying EA [2-4], RAA is present in about 2%-5% of the EA cases [5-7]. Moreover, despite prenatal testing being now widespread and able to diagnose a large proportion of congenital malformations, many authors report a very low rate of preoperative diagnosis of RAA ranging at around 12%-16% [5,7].

The surgical approach in these patients remains controversial. As the traditional approach via right thoracotomy may be complicated by heavily altered vascular anatomy, several authors advocate for a primary left thoracotomy in cases with known or suspected RAA, as this approach offers better exposure of the esophagus and might minimize intraoperative challenges. Conversely, others recommend maintaining a right-sided approach in all cases, arguing that most repairs can be safely performed from the right side, provided that surgeons are prepared for anatomical variations [6,8,9]. The reported outcomes for these patients do not show an obvious advantage for the left-sided approach, nor do they suggest that converting to an anti-lateral procedure is beneficial when RAA is discovered intraoperatively [5,7,8]. According to the systematic review performed by Mentessidou et al. [5], the number of complications like anastomotic leak or stricture or the overall mortality did not differ significantly between the groups approached from either side. However, the authors noticed that the presence of an RAA resulted in unique complications, namely post-operative bleeding from the mobilized aorta, associated with mortality after right-sided repairs. The main issue is that these procedures are being carried out on very small groups of patients, meaning that statistically significant conclusions cannot be drawn.

What might complicate the matter further is the presence of long-gap EA, which is found to occur more often with the cardiovascular defects [6]. Management strategies for long-gap EA vary, ranging from initial gastrostomy with delayed repair to early use of internal traction techniques [11,12]. Internal traction, developed by Foker et al., has gained increasing acceptance as it facilitates early restoration of esophageal continuity while avoiding esophageal replacement procedures [14]. Although the delayed anastomosis is supported by the European Reference Network on Rare Inherited and Congenital Anomalies (ERNICA) consensus, the recent study performed by Borselle et al., in which it was compared with an early anastomosis following internal traction, showed promising results in reducing the time to achieve esophageal continuity [13].

In the present case, despite the suspicion of a right-sided aortic arch, the first operation began on the right side due to the presence of a lower fistula and the surgeons' experience with this approach. However, intraoperative imaging revealed that the patient's anatomy was much more complex than expected, prompting further diagnostic work with cardiac tomography to plan a subsequent operation. Following improvement in the patient's condition after fistula closure, pulmonary valve dilatation, and one of the abnormal arteries being closed, a thoracoscopic procedure could be performed to insert internal traction. Thanks to the visualization of the patient's anatomy, it was decided that a surgical approach from the left side would be more advantageous in this case. According to the method described and developed by Patkowski et al. [12], the authors decided to approach the anastomosis after seven days, which proved beneficial in this patient and allowed for an anastomosis that appeared tight on follow-up contrast studies. Using an ICG-filled balloon is a new approach that did not work well in this case, but the authors intend to refine it, as it may be useful when visualizing esophageal stumps is difficult. Unfortunately, despite the successful surgical treatment, the patient died from the overall burden of multiple congenital defects, which remains the leading cause of mortality in patients with esophageal atresia.

## Conclusions

In conclusion, this case emphasizes the necessity of individualized surgical strategies in patients with EA and associated vascular anomalies, and demonstrates that left-sided surgical access can be a beneficial choice. Preoperative imaging, particularly CT angiography, should be strongly considered to identify detailed vascular anatomy. Furthermore, internal traction techniques represent a valuable option for managing long-gap EA, even in anatomically complex cases.
